# 30-day Mortality Following Revision of Hip Arthroplasty, A Cohort Study Based on the Swedish Perioperative Registry 2017-2022

**DOI:** 10.1177/21514593251355915

**Published:** 2025-06-26

**Authors:** Arvid Ekeberg, Johanna Albert, Olof Sköldenberg, Jon Karlsson, Jan G. Jakobsson

**Affiliations:** 1 Department of Anaesthesia and Intensive Care, Institution for Clinical Sciences, 27106 Karolinska Institute, Stockholm, Sweden; 2 Unit of Orthopaedics, Department of Clinical Sciences at Danderyd Hospital, 27106 Karolinska Institute, Stockholm, Sweden; 3 Department of Orthopaedics, 101051 Sahlgrenska University Hospital and Institute of Clinical Sciences, Gothenburg University, Gothenburg, Sweden

**Keywords:** hip arthroplasty, revision, mortality, all-cause 30-day mortality, perioperative mortality rate, POMR, ASA-class, age

## Abstract

**Introduction:**

In Sweden approximately 9% of hip-arthroplasty require a revision. All-cause 30-day mortality following hip revisions in Sweden is unknown.

**Aim:**

To assess all-cause 30-days mortality among hip-revision in Sweden and whether mortality has changed 2017-2022 based on data from the Swedish perioperative register (SPOR) adjusted for sex, age, ASA-class, indication and fixation technique.

**Method:**

This observational cohort study includes all hip revisions registered in SPOR between 2017 – June 2022 analysing 30-day postoperative mortality rates. Inclusion criteria: age >18 having had a hip arthroplasty revision ICD-10-SE codes NFC. Binary-logistic-regressions estimated odds-ratio (OR) for 30-days mortality over the study period, adjusted for sex, age, ASA-class, indication and fixation technique.

**Result:**

6937 patients were included in the analysis, 3333 females and 3063 males mean-age 73 years with an overall 30-day mortality rate of 1.3%. No significant differences in 30-day mortality were found over the study-period. There was no death within 30-days among patients below 65 years and within ASA-class I. Males had a mortality rate of 1.4% vs females 1.1% (ns.). Adjusted OR was significantly higher for patients with age >80 years OR 9.1 (*P* < 0.001), AS-classes III OR 3.9 and IV 14.9 (*P* < 0.001) infection 3.4 and fracture 9.1 (*P* < 0.001) but fixation technique had not.

**Conclusion:**

The all-cause 30-day mortality in Sweden was 1.3% with minor differences over the study period and between sexes, while high age especially above 80 and ASA-classes III and IV significantly increased the adjusted OR for 30-day mortality. Efforts to further optimize the perioperative care of this patient group are of importance.

## Introduction

Hip arthroplasty is one of the most effective and frequent orthopaedic procedures in the world with over 1 million performed procedures every year. According to the Swedish Arthroplasty Register, approximately 9% of hip arthroplasties need revision surgery with minor changes during recent years.^1^ In a previous study from 2024, the all-cause 30-day mortality associated with primary arthroplasty procedures in Sweden was examined.^1^ The mortality rates in that study differed significantly between the two sub-cohorts defined by indication - hip fracture, and osteoarthritis. As expected, mortality rates were also found to increase with advancing age and higher ASA-class. Additionally, the mortality rates differed depending on whether the indication was elective vs urgent. Revision procedures were, however, excluded from this study. Perioperative mortality rate (POMR) is an important quality indicator of the perioperative course.^2^ Rullan et al published in 2023 a study based on the American College of Surgeons - National Surgical Quality Improvement Program (ACS-NSQIP) database for all patients who underwent revision total hip arthroplasty (THA) between 2011 and 2019, where they assessed the all-cause 30-day mortality and especially the impact of age and comorbidities.^3^ They found an overall POMR of 0.69% and with a major impact related to patient age and comorbidity. There is no recent study assessing the all-cause 30-day mortality following revision of hip arthroplasty in Sweden available. Although the most common cause for revision – aseptic loosening – is steadily decreasing. The growing number of hip arthroplasties in Sweden will inevitably lead to increased need for revision procedures in patients at higher age and ASA-classes. It is subsequently essential to analysing 30-day mortality, perioperative mortality rate (POMR), and the impact of high age and ASA-class. The Swedish perioperative register (SPOR^2^) was started in 2012 to provide opportunity for quality control of perioperative care in Sweden.^4^

This study aims to assesses the all-cause 30-day perioperative mortality rate (POMR), in relation to hip revision in Sweden and whether annual the POMR has changed between 2017 and 2022, based on data from the Swedish perioperative register, SPOR, and further to assess the impact of sex, age, and ASA-class.

The hypothesis was that the overall 30-day mortality would have decreased taking the general improvements in perioperative care during recent years including increasing the enhanced recovery after surgery bundle.

## Materials and Methods

### Study Design

This is a registry-based cohort study, utilizing data collected from the Swedish Patient Registry (SPOR). The reporting of this study conforms to the Strengthening the Reporting of Observational Studies in Epidemiology *(STROBE)* Statement: guidelines for reporting observational studie^3^.

### Data Source

All hip revision procedures registered in SPOR between 2017 – June 30^th^ 2022, in patients above 18-years and ASA-classes I-V having undergone ICD-codes NFC procedure were retrieved from the Swedish perioperative quality of care register SPOR . Patients with missing data on mortality, age, ASA, procedural codes, or errors with registered days of death were excluded. For patients with multiple procedures, only the most recent was included.

## Variables

Variables included in the analysis dataset were patient characteristics, age, sex, and ASA-class. Perioperative data: indication, arthroplasty technique, main anaesthetic technique, and all mortality within 90-days. Categorization of data; Hip revision procedures were divided using ICD-10-SE codes, NFC09-NFC99 (see appendix ICD-codes). Age was categorized into decades and further into 2 groups of 18-80 and above 80 (80+). Sex was sorted as male or female. ASA-class was categorized as I+II, and III, IV and V, respectively. The indication for surgery was classified associated to the ICD-codes for osteoarthritis M16.0-M16.9, fractures as ICD-codes S720-S72.9 and other diagnoses grouped as “others”. Anaesthesia techniques were classified as neuraxial anaesthesia with or without sedation and general inhaled anaesthesia. Mortality was based on the coordination of SPOR and the Swedish death register, which is done automatically by SPOR.

Primary outcome was all-cause 30-day mortality, perioperative mortality rate as defined by the Utstein definition number of deceased divided by all procedures.^5^

Exposure was the change in POMR over the 6-year period studied, explicitly the extreme years, 2017 and 2022, were carefully studied.

### Statistical Analysis

Continuous variables, e.g. age, are presented as mean and standards deviation (SD). Categorical variables, age-groups and ASA-class are presented as number and percentages (%). The distribution of continuous numerical variables was analysed by reviewing the data as a histogram, conducted by a statistician at the Karolinska Institute. Comparison between groups was performed with independent t-test, ANOVA for numerical continuous variables and Chi-2 test for categorical data. A binary logistic regression was conducted using the odds ratio (OR) to assess all-cause 30-day mortality, unadjusted and adjusted for age, ASA-class, sex, and indication. A *P*-value <0.05 was considered statistically significant. All data was handled in Microsoft 365 Excel, and statistical analyses were conducted using the SPSS 28 software.

## Results

**Figure 1.**
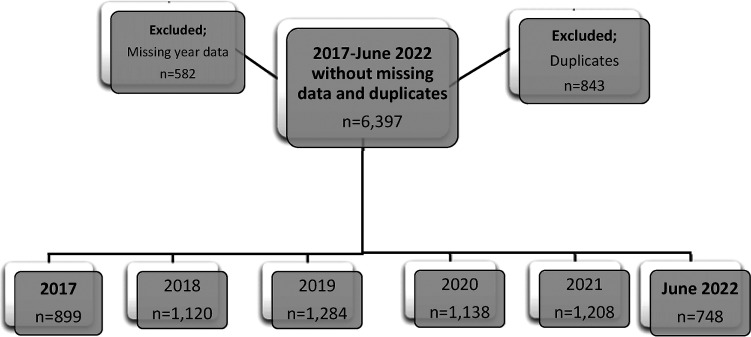
Flow Chart Describing patient inclusion and Exclusion

A flowchart that describes the study population and its dividends during the intervention years is presented in Figure 1.

Overall, 6397 patients, 3063 males and 3333 females, (one missing value) with a mean age of 73 ± 11 years old were included in the analysis. The patients’ characteristics over the study period are presented in Table 1. No significant differences in sex distribution were observed over the study period (*P* = 0.78), with females comprising a numerically higher proportion throughout the study period (0.8% to 5.8%), except in 2019, where males were slightly overrepresented by 0.2%. Mean age was consistent over the period with an overall mean age of 73 ± 11 years, the females being significantly older than male patients 74 ± 11 vs 72 ± 11 (*P* < 0.001). Similarly, no change in mean age was observed. Overall, 5031 (79%) of patients were above 65 years of age and 1743 (27%) over 80 years. 3205 (50%) of patients were ASA-class II, 2465 (39%) ASA-class III, 488 (8%) ASA-class I, and 179 (3%) ASA-class IV, with minimal variation over the period studied. There were no ASA-class V patients. Males were classified in a significantly higher proportion of ASA-classes III and IV (*P* = 0.047). The most common indication was mechanical issues (loosening), (56%), followed by infections (15%) and fractures (9%). The proportion of indications varied statistically significant during the study period (*P* = 0.002). World Health Organisation safe surgery checklist was used in overall 83% of procedures, with limited variation of the period studied (Table 1).

**Table 1.** Patients Characteristics for Hi Revisions Over the 6-Year Period Studied Based on SPOR

	2017 n = 899	2018 n = 1120	2019 n = 1284	2020 n = 1138	2021 n = 1208	2022 – June n = 748	All n = 6397	*P*-value
Age years ± SD	73 ± 11	73 ± 11	74 ± 11	73 ± 12	73 ± 11	74 ± 11	73 ± 11	ns
Age class; 18-65/66-80/80+	209/461/229	2347607/279	234/677/364	255/556/327	285/593/330	140/394/214	**1366/3288/1743**	ns
Female/male No of pat *%*	485/414 *54/46*	590/530 *53/47*	650/634 *51/49*	601/536 *53/47*	626/582 *52/48*	381/367 *51/49*	**3333/3063** ***52/48***	ns
ASA-class I/II/III/IV No of pat	76/458/320/27	103/574/404/28	92/636/509/35	94/570/437/30	77/595/496/28	46/372/299/22	**488/3205** (*58%*)**/2465/170** (*42%*)	ns
**Indication** No of pat*%*								**0.002**
Mechanical loosening	512	640	721	613	623	449	**3558** ***56***	
Infection	109	147	176	167	216	121	**936** ***15***	
Fracture	79	79	110	104	129	68	**569** ***9***	
Other and missing	199	254	277	254	240	110	**1334** ***21***	

SD standard deviation, No of pat number of patients, ASA-class American Society for Anaesthesiology physical status, ns non-significant, SPOR Swedish Perioprative Register. All analysis are assess by 2-sided tests. Bold presents statistically significant *p*-value *p* < 0.05 and the entire cohort values.

Perioperative observations are presented in Table 2. Time events were most consistent over the study period. Mean duration of surgery was 2 hours and 34 minutes, mean duration of anaesthesia 3 hours and 57 minutes and mean recovery room stay 6 hours and 20 minutes with a huge range between 46 minutes up to 23 hours. Anaesthesia had several missing and incomplete information in the registry, neuraxial anaesthesia with or without cover sedation was the predominant anaesthetic technique used in 66% of procedures, general anaesthesia with or without loco-regional anaesthesia was used in 34% of procedures. Cemented arthroplasty was the most used procedure (38.2%) followed by cementless (34.6%) and hybrid techniques (27.2%). There was a significant shift in technique with decreased use of cemented arthroplasties and increased use of hybrid and cementless arthroplasties (*P* = 0.002) (Table 2).

**Table 2.** Perioperative Observations for Hip Revision Procedures Over the 6-Year Period Studied Base on SPOR Data

	2017 n = 899	2018 n = 1120	2019 n = 1284	2020 n = 1,,138	2021 n = 1208	2022 – June n = 748	All n = 6397	*P*-value
Duration of surgery min. ± SD	154 ± 73	153 ± 72	153 ± 73	153 ± 71	154 ± 70	154 ± 72	**154 ± 72**	ns
Duration of anaesthesia min. ± SD	241 ± 83	237 ± 83	237 ± 85	235 ± 84	236 ± 81	237 ± 82	**237 ± 82**	ns
Arthroplasty								
Cemented	339	413	411	372	372	236	**2150**	
Cement-less	276	309	416	326	380	236	**1943**	
Hybrid	183	275	295	300	304	171	**1528**	
Duration of recovery room stay min. ± SD	397 ± 332	403 ± 339	399 ± 329	360 ± 302	346 ± 332	358 ± 298	**374 ± 324**	ns
WHO Checklist used Yes/No No of pat *%*	707/192 *79/21*	905/215 *81/19*	1025/259 *80/20*	971/167 *85/15*	1045/163 *86/14*	659/89 *88/12*	**5312/1085** ***83/17***	ns

SD standard deviation, No of pat number of patients, ns non-significant, SPOR Swedish Perioprative Register, all analysis are assess by 2-sided tests. Bold presents statistically significant *p*-value *p* < 0.05 and the entire cohort values.

### Outcomes

#### Perioperative Mortality Findings of the Population over Study Period

**Figure 2.**
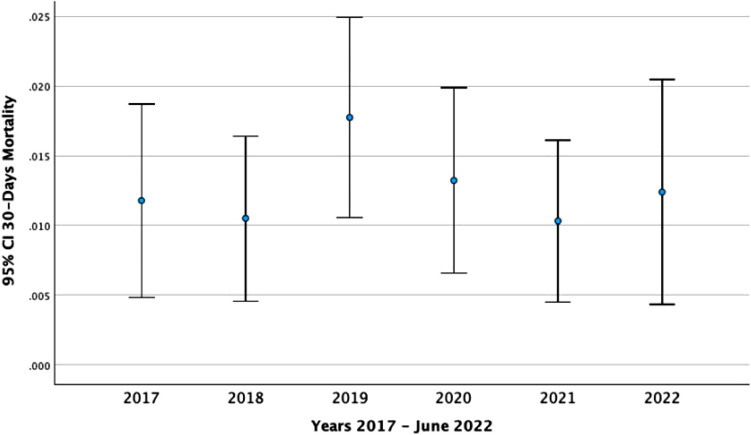
30-day Mortality Over the Years 2017 to 2022 Presented With a 95% Confidence Interval. Perioperative Mortality Rate in Accordance Too the Utstein Revised Definition, Proportion of Deceased Divided by Annual Number of Procedures in Percent and 95%CI, Confidence Interval

The perioperative mortality rates over the study period are presented in Table 3. The overall-cause 30-day mortality was 1.3%. There were no significant differences in the annual 30-days perioperative mortality rate over the study period (*P* = 0.678). POMR varied between 1.0% in 2021 and 1.8% in 2019. Likewise, no significant differences were observed in deceased within day of surgery, 7-days, and 90-days between years studied. Annual POMR including 95% CI is also displayed in Figure 2.

**Table 3.** Perioperative Mortality Rates Over Study Period Number of Deceased, POMR-Percent, and 95 Percent Confidence Interval (n, Column, %, 95% CI) for Hip Arthroplasty Revision Based on SPOR Data

	2017 n = 899	2018 n = 1120	2019 n = 1284	2020 n = 1138	2021 n = 1208	2022 – June n = 748	All n = 6397	*P*-value
Deceased day of surgery	2 (0.02)	1 (0.08)	1 (0.07)	1 (0.09)	1 (0.09)	1 (0.13)	**6 (0.09)** **0.04-0.19**	
Deceased within 7-days	3 (0.32)	6 (0.52)	3 (0.23)	5 (0.44)	1 (0.09)	4 (0.55)	**22 (0.34)** **0.22-0.51**	*P* = 0.74
Deceased within 30-days n (%), 95%CI	11 (1.2)	12 (1.1)	23 (1.8)	15 (1.3)	12 (1.0)	9 (1.2)	**82 (1.28) 1.03-1.58**	*P* = 0.60
Deceased within 90-days	29 (3.2)	29 (2.6)	45 (3.5)	38 (3.3)	32 (2.6)	21 (2.8)	**194 (3.03)** **2.63-3.47**	*P* = 0.66

Abbreviations: POMR – Perioperative Mortality Rate, CI – Confidence interval, SPOR Swedish Perioprative Register, all analysis are assess by 2-sided tests. Bold presents statistically significant *p*-value *p* < 0.05 and the entire cohort values.

**Figure 3.**
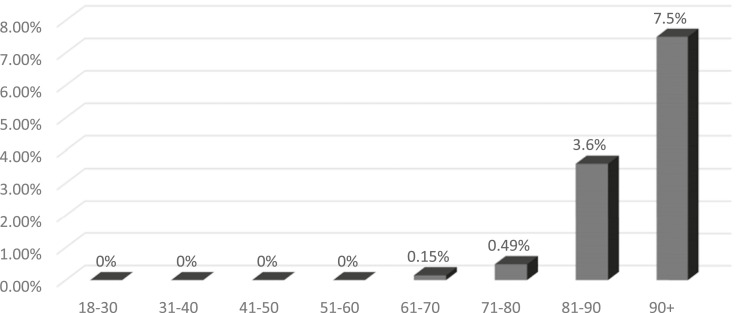
All-Cause 30-Day Mortality, Age, Years in Decades, Upper Limit of Age is Presented

The impact of age and ASA-class is further visualised in the landscape diagram (see appendix). There were no patients in ASA-class I with an all cause 30-day mortality, and none below 65 years of age, while the highest proportion of deceased was found in patients between 90+ years of age and ASA-class IV. The all-cause 30-day mortality for the cohort classified by decade of age is presented in Figure 3. Mortality rate increased dramatically up to 7.5% for the age class 90+ above of age. There were merely 3 patients having a revision above 100 years and none of these died during the early postoperative follow-up.

All cause 30-day mortality differed significantly between indications (*P* < 0.001) mechanical loosening was associated with a 0.6% mortality, infections 2.0% and fractures 5.1%. Available data related to the anaesthetic technique did not indicate any impact on mortality.

#### Binary Logistic Regression Examining the Impact of Age, ASA, Sex, and Indication

The results from the binary logistic regression analysis, assessing the impact of the intervention, change over study period adjusted for age, ASA-class, sex, indication and fixation technique impact on the all-cause 30-day mortality rate, POMR, are presented in Table 4. There were no significant differences in the OR between study years in the regression model adjusted for patient characteristics and indication for revision. The minor increase in OR for males did not reach statistical significance. Age above 80 years had a significantly higher adjusted OR as did ASA-classes III and IV in the adjusted model. Patient having revision due to infection and fracture did also have significantly higher OR for all-cause mortality within 30 days. The fixation technique did not have any significant impact on early mortality when adjusted for patient factors (sex, age and ASA-class) and indication.

**Table 4.** Binary Logistic Regression Assesses OR for All-Cause 30-Day Mortality Among Hip Arthroplasty Revisions in Sweden Over the Study Period Adjusted for Sex, Age, ASA-Class, Indication and Technique of Fixation Based on SPOR Data

	Odds ratio	95% C.I.		*P*-value
		Lower	Upper	
Year 2017	-			
Year 2018	1.5	0.5	3.9	0.5
Year 2019	1.8	0.7	4.5	0.2
Year 2020	1.3	0.5	3.4	0.6
Year 2021	0.9	0.3	2.5	0.9
Year 2022	1.2	0.4	3.2	0.8
Age-class 18-80	-			
Age-class 80+	9.1	4.6	18	**0.001**
ASA-class I/II	-			
ASA-class III	3.9	1.8	8.1	**0.001**
ASA-class IV	14.9	6.0	36.6	**0.001**
Male	1.6	0.9	2.6	0.08
Mechanical loosening	-			
Infection	3.4	1.8	6.4	**0.001**
Fracture	9.1	4.6	18.0	**0.001**
Cemented prosthesis	ref			
Cementless	0.8	0.4	1.6	0.5
Hybrid	0.9	0.4	1.9	0.9

CI - confidence interval, ASA - America Society for Anesthesiology physical status, SPOR Swedish Perioprative Register. Bold presents statistically significant *p*-value *p* < 0.05 and the entire cohort values.

## Discussion

This quality register based (SPOR) study aimed to assess the all-cause 30-day mortality following hip revision procedure in Sweden and if adjusted annual perioperative mortality rate has changed during the period 2017 and June 2022. Moreover, to assess the crude annual POMR and whether adjusting for sex, age, and ASA had impact on the annual POMR odds ratio.

The study showed an all-cause 30-day mortality of 1.3% (n = 82) for the total study population (n = 6397). There were no significant differences in the 30-day mortality neither over the period nor between the extreme years 2022 (January 1st to June 31^st^) and 2017 when analysed separately (*P* = 0.901). The annual OR for POMR over the study period was further not impacted by adjusting for sex, higher age, and ASA class. There were no persons deceased within 30-days for any ASA-class I patients below 65 years of age. There was an increased mortality as expected for both increasing age and higher ASA-classes, while sex did not impact the 30-day mortality. 30-day mortality was 3.6% among patients between 81 and 90 years and increased to 7.5% in patients above 90 years of age. Moreover, infection and fracture as an indication was correlated with significantly higher OR for all-cause mortality within 30 days.

### POMR - 30-Day Mortality

Although hip revision surgery inherently carries risks, the findings of the present study show a relatively low 30-day mortality rate. There is sparse data around all-cause 30-day mortality following revision procedures. Rullán et al, analysed revision of total hip arthroplasty performed between 2011 and 2019 based on US register data, and found a mortality lower than that observed in the present study.^3^ However, when mortality was categorized in deceased according to age decades, the mortality rate for was similar to the present study. Notably, while the US study cohort showed patients deceased in ages below 60, no fatalities occurred in patients below 65 years in the Swedish cohort. Lamb et. al., conducted a meta-analysis assessing mortality following hip arthroplasty revision due to femoral fractures, reporting a 30-day mortality rate of 3.3%, slightly lower than the 5% observed in the present study.^6^ However, the 95%CI for the 30-day mortality overlaps the POMR in the present study, 95% CI 2.0% to 5.0%. Laughlin et. al., published in 2021 a single centre study assessing mortality rates follow hip revision surgery between 2012 and 2018 in 596 patients.^7^ They found an overall mortality of 3.38 per 1000 procedures but with a 95% confidence interval well covering the present study. The mean age of their patients was 62 years compared with 73 years in the present study. Dagneaux et al studied mortality associated with aseptic revision arthroplasty in patients above 90 years and identified a 90-days mortality of 8%,^8^ in line with our findings. There is a study based on the Swedish arthroplasty register assessing mortality after total hip prosthetic joint infection between 1998 and 2017 studying mortality up to 4 years after surgery showing similar results as ours for the 30-day mortality. That study found also that that fatal outcomes are mainly associated with by the comorbidities.^9^ A most recent study from US showed that early postoperative infections are important risk factor for early death.^10^ The SPOR-based study by Magnusson et. al., which examined early all-cause mortality in patients undergoing primary hip arthroplasty in Sweden, showed a higher 30-day mortality rate of 2.8%.^1^ Gremillet and Jakobsson, demonstrated a 30-day mortality of 7.7% in patients undergoing acute hip fracture surgery in Sweden 2016 and 2017 based on SPOR data.^11^ Thus, it can be concluded that the overall early mortality rate is not by any means alarming. One must also consider that there was no death in patients below 65 years of age, the majority of death were seen in patients age above 80 years.

### Patients’ Characteristics and Impact on POMR

In the present study, there was a non-significant, but high percentage of male deceased within 30-days as compared with females, who counted for 52.3% of the total study population. These findings align with those of Gremillet and Jakobsson, who reported a significant correlation between male sex and an increased likelihood of mortality within 30-days following ankle fractures, compared with females.^10^ The mean age was significantly higher among deceased (*P* < 0.001) and the proportion of ASA-class III and IV was also significantly greater among the deceased patients (*P* < 0.001). The mean age among alive patients was nearly 7 years lower as compared with the deceased. Similar findings were reported by Rullán et al. who also highlighted the dramatic impact of age on the POMR following hip arthroplasty.^3^ Additionally, the mean age in the present study was more than 10 years higher than that reported by Laughlin et al^7^ However, the findings in terms of age and ASA-class distribution were comparable to those reported by Jayasinghe et al^12^ and Resl et al^13^. The study do shows in the regression analysis a significantly higher adjusted OR for patients aged 81 and older and those with elevated ASA scores. Similar findings were reported in the study by Magnusson et al, who identified both higher age and higher ASA scores as contributing factors to elevated all-cause 30-day mortality following primary hip arthroplasty.^1^ A previous study by Ferguson et al. has likewise demonstrated that advanced age and higher ASA-classes is associated with an increased risk of complications and finally mortality in patients undergoing hip replacement.^14^ The SPOR registry merely collect age and ASA-class. Still, these most commonly collected patients’ characteristics do provide important information about the odds for early mortality. Whether further information such as fragility score and the Charlson comorbidity index^4^ could even further enhance risk stratification cannot be determined from the present study.

### Fixation and Anaesthetic Technique Impact on POMR

Mechanical loosening was the most common indication in line with Jayasinghe.^11^ However, both infection and fracture indications were associated with significantly higher OR for early mortality compared with mechanical loosening. This finding is in line with the study by Laughlin et al, who assessed mortality up to 2 years.^7^ Fixation technique shifted during the study period with an increased use of cementless and hybrid techniques, but fixation technique did not impact mortality in the adjusted model in the present study. Magnusson et al. found that hybrid fixation was associated with a lower OR for the all-cause 30-day mortality following primary hip replacement in Sweden during a similar study period, but when analysis by indication was done, this lower OR was no longer seen.^1^ A Norwegian hip arthroplasty register based study published in 2020, found among 79,557 patients, including primary THA patients no differences in short-term, and long-term mortality rates after primary hip arthroplasty.^15^ In the present study, there was no significant impact of fixation technique in the adjusted model, taking patients characteristics and indications into account. A recent major register-based study from US found a surprisingly four times higher 30-day mortality for femoral as compared to acetabular re-arthroplasty.^16^ The present study did, unfortunately not, include more technical details around the surgical procedure.

### Assessment of Findings

We are not able to provide any firm cause for the overall reassuringly low and stable low 30-day mortality found in this observational register-based study. It is still obvious, based on the findings from the present study, that the “patient at risk” for early mortality is the ASA III-IV patient in higher age. The benefit vs risk must indeed be assessed for the geriatric ASA III-IV patient. Further, prehabilitation should be consider to as far as possible reduce risk and facilitate recovery.^17^ Our low mortality may at least to some extent be associated with that majority of revisions are performed in university centres with rather high annual number of revision procedures.^5^ Determining indications and choosing techniques can be challenging, and intraoperative complications and unexpected findings during revision surgery are not uncommon. Thus, it is important to have experienced and specially trained staff, as well as access to special instruments, bone bank, and a sufficiently large range of implants. The extensive national implementation of Enhanced Recovery after Surgery bundle for the hip arthroplasty has most certainly also had impact on mortality rate.^18^

This study is based on a nationwide registry, including a robust dataset, spanning over a six-year period, from the Swedish Perioperative Register (SPOR).^4^ There are, however, several limitations to this study. There is a lack of reconciliation between national quality registers. The numbers procedures included are lower as compared to the Swedish Arthroplasty register^1,^^6^ thus there is a registration bias. The mean age of the cohort, the proportion of patients below 65 years in our cohort is 21 % similar vs 22 % in the arthroplasty register. The proportion of ASA 1-2 is also similar for the study cohort 58% and in the arthroplasty register 53% for revisions. The patients’ characteristics suggest however that the cohort is a reasonable sample of revision procedures in Sweden.

While age, sex, and ASA classification are relevant and commonly assessed patient factors associated with mortality, they do not provide in-depth detailed information about comorbidities. Including additional variables, such as fragility scores^7^ or the Charlson Comorbidity Index, could offer more nuanced insights into patients’ health status. It is also a major limitation that the register does not provide information about the cause of death, thus limiting the possibilities to fine tune interventions for improvement. The limited information about further details around patients’ comorbidity, potential selection bias and postoperative course following discharge from recovery unit must be acknowledged.

As addressed before, no more in-depth assessment of surgical aspects, surgical techniques and arthroplasty types are included in the analysis. An additional limitation to this study is the number of missing data, which reduces the study’s overall validity and limits its external applicability. The registry data used in this study is collected solely in Sweden with a tax-funded health care system, which restricts the findings from being generalized or compared with outcomes from other countries.

## Conclusion

This study found an all-cause 30-day mortality rate of 1.3% following revision of hip arthroplasty in Sweden with no change over the study period 2017 to 2022. There was no death among patients in the age category below 65 years and with an ASA-class I. Increasing age especially above 80 years, ASA-classes III and IV and fracture indication did dramatically increase the Odds Ratio for mortality. Further efforts to optimize perioperative care for the geriatric as well as ASA III-IV patients with a need for hip arthroplasty revision because of infection or fracture is of great importance.

## Data Availability

The data that support the findings of this study are available on request from the corresponding author. The data are not publicly available due to privacy or ethical restrictions. *

## Notes

1. https://slr.registercentrum.se/om-registret/arsrapporter/p/r1VTtHNvu, https://efaidnbmnnnibpcajpcglclefindmkaj/https://registercentrum.blob.core.windows.net/sar/r/Swedish-Arthroplasty-Register-Annual-report-2024-ENG-CHsgLK06p.pdf.

2. SPOR – Svenskt Perioperativt Register – Swedish Perioperative Quality register.

3. https://www.strobe-statement.org/.

4. https://www.mdcalc.com/calc/3917/charlson-comorbidity-index-cci.

5. https://efaidnbmnnnibpcajpcglclefindmkaj/https://registercentrum.blob.core.windows.net/sar/r/Swedish-Arthroplasty-Register-Annual-report-2024-ENG-CHsgLK06p.pdf.

6. The Swedish Arthroplasty Register.

7. https://www.dal.ca/sites/gmr/our-tools/clinical-frailty-scale.html.
